# Systemic Inflammation and Disruption of the Local Microenvironment Compromise Muscle Regeneration: Critical Pathogenesis of Autoimmune-Associated Sarcopenia

**DOI:** 10.2196/64456

**Published:** 2025-05-23

**Authors:** Yingjuan Zhang, Qingqian Wu, Yi Wang, Qingyan Chen, Shuang Han, Bei Li, Qingwen Zhao, Qianzhuo Wang, Yule Wang, Yue Gao

**Affiliations:** 1 Department of Geriatrics, Zhejiang Key Laboratory of Traditional Chinese Medicine for the Prevention and Treatment of Senile Chronic Diseases, Affiliated Hangzhou First People’s Hospital, School of Medicine, Westlake University, 261 Huansha Road, Shangcheng District, Hangzhou, 310006, China, 86 13706511908; 2 Yixing Center for Disease Control and Prevention, Yixing, Jiangsu Province, China; 3 Fourth Clinical School of Medicine, Zhejiang Chinese Medical University, Hangzhou, China

**Keywords:** sarcopenia, immune diseases, type 1 diabetes mellitus, rheumatoid arthritis, inflammatory bowel disease

## Abstract

Sarcopenia is defined by age-related reductions in muscle mass, strength, and physiological function, and it is especially prevalent among individuals with autoimmune diseases. Autoimmune disorders, characterized by immune dysregulation, cause systemic inflammation and damage to multiple tissues through unregulated immune activity. Research indicates that autoimmune diseases negatively impact skeletal muscle functions and may worsen the progression of sarcopenia. This viewpoint comprehensively discusses the pathogenesis and potential mechanism of sarcopenia in 3 autoimmune diseases: inflammatory bowel disease, rheumatoid arthritis, and type 1 diabetes mellitus. Mechanistically, chronic immune microenvironment alterations induce compartment-specific redistribution of leukocyte subsets and cytokine networks. These perturbations disrupt critical signaling pathways governing muscle protein synthesis, satellite cell activation, and mitochondrial bioenergetics, leading to impaired regeneration and accelerated sarcopenia progression. By delineating shared and distinct pathomechanisms across these models, this analysis reframes our understanding of immune-mediated muscle wasting. Beyond mechanistic insights, it establishes a translational framework for targeted therapies and highlights emerging research directions bridging immunology and age-related musculoskeletal decline.

## Introduction

Sarcopenia is an age-related syndrome characterized by progressive and systemic loss of skeletal muscle mass and strength [1]. The decline of skeletal muscle function caused by sarcopenia has aroused widespread concern [2]. Maintaining skeletal muscle mass and integrity is essential for the proper functioning of the musculoskeletal system and for the effective absorption and storage of nutrients [3]. Sarcopenia has a significant impact on general health, with numerous important effects. It is closely related to the development of exercise-related diseases, such as falls, fractures, and disabilities [4]. Additionally, it is also associated with adverse outcomes such as cardiovascular diseases [5], metabolic diseases, and even death [6]. However, the exact cause of primary sarcopenia is not fully understood yet. Recent studies suggested that cellular aging [7], mitochondrial disorder [8], decreased physical activity [9], and excessive caloric intake could accelerate the progression of sarcopenia. Moreover, an imbalance of oxygen in the body may contribute to the development of sarcopenia as well [10].

Sarcopenia afflicts not only the older people but also the patients with immune diseases. Studies have shown that immune diseases such as type 1 diabetes mellitus (T1DM), rheumatoid arthritis (RA), inflammatory bowel disease (IBD), spinal arthritis, and systemic sclerosis are often associated with sarcopenia [11]. Autoimmune diseases combined with sarcopenia are closely related to adverse clinical outcomes, poor effects of treatment and prognosis, and low quality of life [12]. Current research efforts have concentrated on the high prevalence of sarcopenia among autoimmune disease patients and its associated adverse prognosis. Studying the relationship between sarcopenia and autoimmune diseases is crucial, as is clarifying the incidence and pathogenesis of sarcopenia linked to immune disorders. Immune disorders regulate key cascades like JAK/STAT (Janus kinase/signal transducers and activators of transcription) by influencing immune cells, profoundly impacting muscle protein synthesis and metabolism. This is a crucial factor in the pathogenesis of autoimmune diseases accompanied by muscular atrophy. Previous studies have focused on the differences in performance between healthy and sick individuals through case-control analysis. However, our comprehensive and in-depth analysis shows that focusing on immune cells and the related factors released and exploring the pathogenesis of diseases based on this, to find effective targeted therapies, will become a new and promising direction for the study of autoimmune diseases and sarcopenia. The elucidation of these underlying mechanisms may pave the way for novel research avenues in understanding the pathophysiology of sarcopenia associated with autoimmune disorders, while simultaneously offering crucial mechanistic insights to inform the development of targeted therapeutic interventions with enhanced precision. This study aims to provide a theoretical foundation and new avenues for future research.

## Sarcopenia

**Figure 1.**
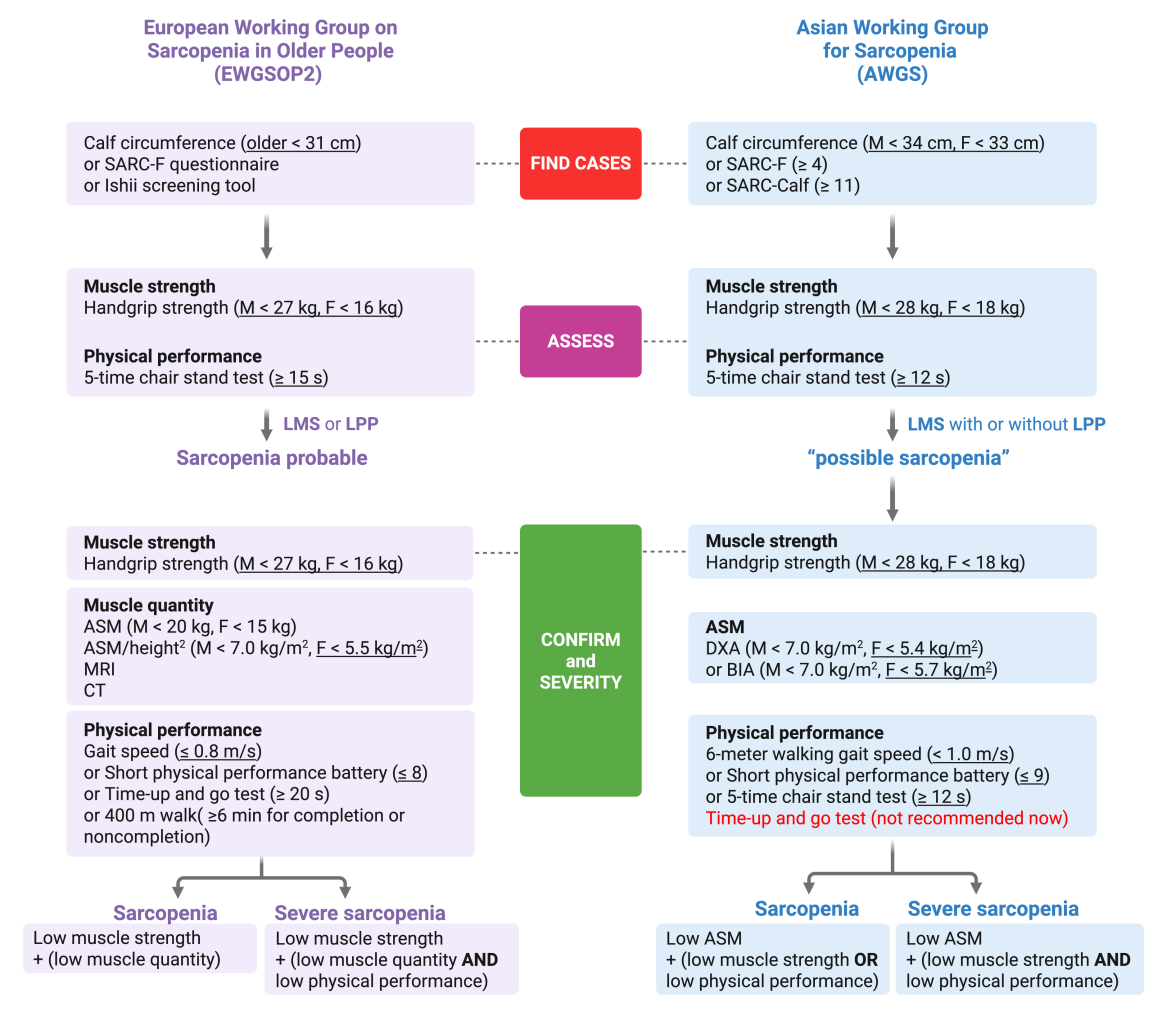
Simple algorithms for Europeans and Asians to diagnose sarcopenia in clinical practice respectively. ASM: appendicular skeletal muscle mass; BIA: bioelectrical impedance analysis; CT: computed tomography; DXA: dual-energy x-ray absorptiometry; F: female; LMS: low muscle strength; LPP: low physical performance; M: male; MRI: magnetic resonance imaging; SARC-Calf: SARC-F plus calf circumference (score of ≥11 is termed as sarcopenia); SARC-F: strength, assistance in walking, rising from a chair, climbing stairs, and falls (score of ≥4 is termed as sarcopenia).

In 1989, Irwin Rosenberg first described sarcopenia as the loss of muscle mass that occurs with aging [13]. With the deepening of research, sarcopenia is generally recognized as a progressive and systemic skeletal muscle disease manifested by accelerated loss of muscle mass and function [14]. The European Working Group on Sarcopenia in Older People 2 updated the definition of sarcopenia to account for muscle mass, muscle strength, and motor function performance for the first time [13]. The European Working Group on Sarcopenia in Older People 2 suggests that sarcopenia may be present when muscle strength is insufficient, and can be diagnosed when low muscle quantity or quality exist simultaneously [13]. Moreover, when the patient has low muscle mass, low muscle strength, and weak motor function at the same time, severe sarcopenia can be diagnosed [15]. Applying Western diagnosis criteria to Asian people may not be adequate. For special considerations such as different body sizes, fat distribution, and physical activity from the Asians, the Asia Working Group for Sarcopenia proposed and revised an algorithm for diagnosing sarcopenia based on the Asian data [1,16] (Figure 1).

The prevalence of sarcopenia in older individuals within community health care settings has been reported to reach up to 29% [16]. Aging changes the homeostasis of skeletal muscle and disturbs the balance between anabolic and catabolic processes on the protein production pathway [16]. Sarcopenia is characterized by a decrease in the size and number of satellite cells and the atrophy of type 2 muscle fibers. Sarcopenia is distinct from malnutrition and cachexia, although each exhibits interrelated pathophysiology leading to different magnitudes of “wasting” and susceptibility to cardiovascular events (Table 1) [2].

Diagnosing sarcopenia requires a combined measurement of muscle mass, strength, and physical performance [17] (Figure 1). At present, imaging methods, such as computed tomography, dual-energy x-ray absorptiometry (DXA), and magnetic resonance imaging are used for muscle mass detection [18]. However, those instruments are expensive, time-consuming, and laborious. Besides, computed tomography detection has a certain level of radioactivity, which limits its application in clinical practice and population research [19]. DXA can distinguish adipose tissue from other soft tissues and measure the volume of tissues and organs accurately, so it is suitable for different populations and is considered the “gold standard” for measuring muscle mass [20]. Owing to the characteristics of fast, convenient, and low radioactivity, DXA is also frequently used for body composition detection. However, DXA has the limitations of not assessing muscle quality (ie, muscle fat infiltration), and DXA measurements may be influenced by the patient’s hydration status [21]. The machine requires a large space and is inconvenient to move away, making DXA not an ideal measurement for large-scale clinical trials of sarcopenia, bedside assessments, or community health screening work [18]. Bioelectrical impedance analysis (BIA) is another measurement for muscle mass detection. The principle underlying BIA is that different body compositions at the tissue-organ level exhibit varying electrical conductivity, with skeletal muscle demonstrating good conductivity and adipose tissue showing poor conductivity. This enables BIA to estimate the quantity of body fat and skeletal muscle mass [22]. Comparatively speaking, except inexpensive, BIA is a portable device that can easily perform bedside diagnostics as well. Therefore, BIA is frequently used in many types of studies. As BIA equations and cut-off values are population and device-specific, results can vary between devices and different populations [23]. Moreover, studies showed that the mass of hospitalized older patients was overestimated [24]. Nevertheless, the results of BIA would also be affected by the placement of electrodes, so it is necessary to combine grip strength and walking speed detection to evaluate the occurrence of sarcopenia [25].

Sarcopenia increases the risk of falls, fractures, and death. When combined with other diseases, it can hinder recovery after hospitalization, prolong prognosis recovery time, and increase the economic burden on patients [26]. Previous studies suggested that sarcopenia and osteoporosis interacted with each other. Compared with healthy people, individuals with sarcopenia have a heightened susceptibility to developing osteoporosis. Conversely, individuals experiencing severe osteoporosis often undergo substantial loss of skeletal muscle mass, thereby increasing their vulnerability to falls and fractures [27]. Sarcopenia is known to adversely affect the outcomes of patients with various forms of cancer. Apart from what is mentioned above, there exists an interaction between sarcopenia and immune diseases. The complication of sarcopenia greatly increases the incidence; mortality; and prognosis of diabetes, arthritis, and IBD.

**Table 1.** Differences between sarcopenia, cachexia, and malnutrition.

	Sarcopenia	Cachexia	Malnutrition
Clinical features	Loss of muscle strength and muscle mass	Weight loss with loss of muscle mass	Weight loss
Functional impairment	Indicate severity	+++^a^	−^b^
Mechanism	Age-related, pathologic	Pathologic	Inadequate caloric intake, malabsorption
Inflammation	+/−^c^	+++	−
Anorexia	+/−	++^d^	+/−
Fat mass	+/–	Decreased	Decreased
Protein degradation	+/−	+++	+^e^

a +++: high, a higher degree or intensity of a feature or symptom in the disease.

b −: absence of the characteristic/symptom indicates the absence or absence of a characteristic, symptom, or mechanism.

c +/–: variable presence or inconsistency across conditions.

d ++: moderate, the presence or intensity of a characteristic or symptom in the disease.

e +: low, a feature or symptom that is less present or intense in the disease.

## Autoimmune Diseases

**Figure 2.**
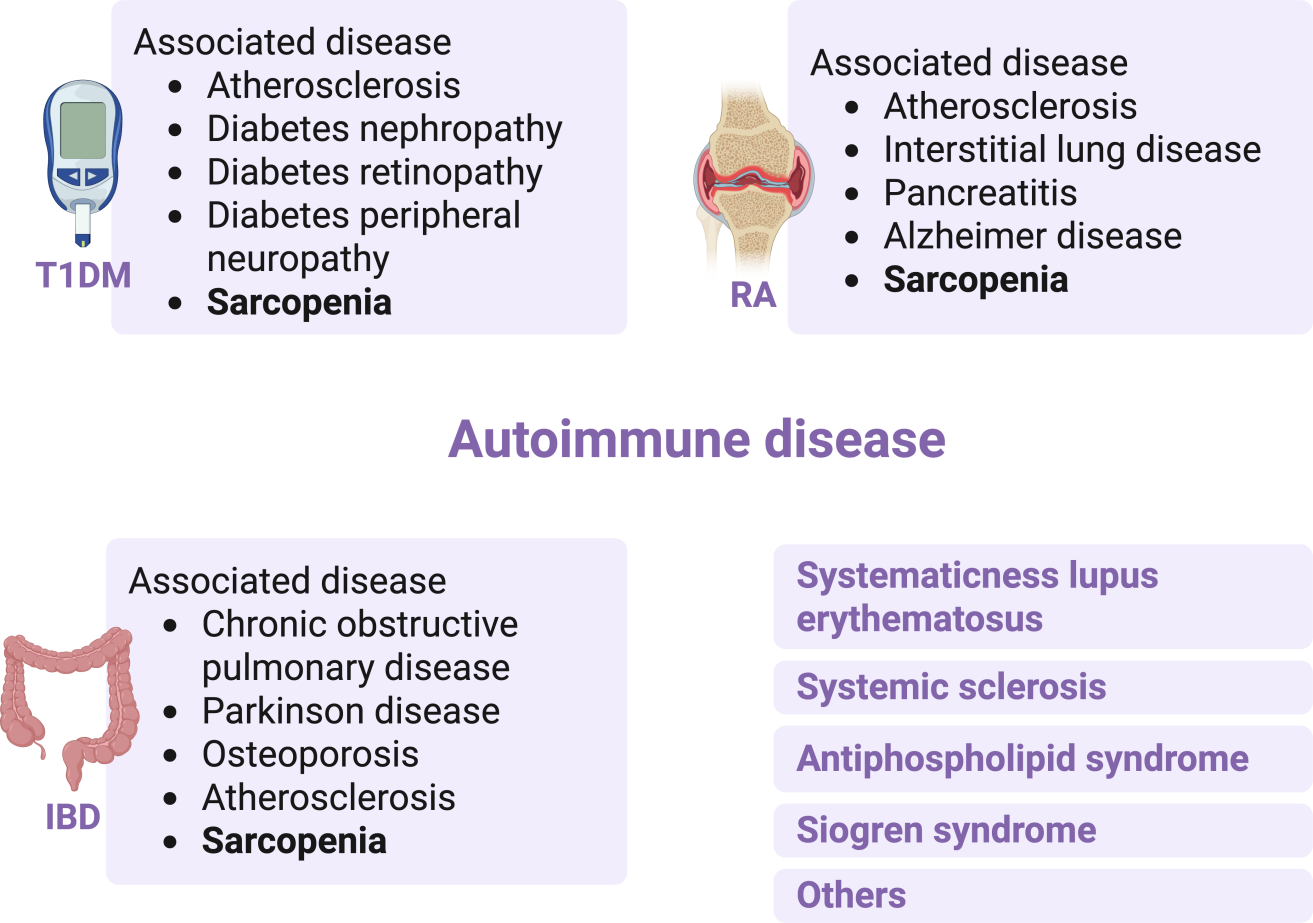
The associated disease of autoimmune diseases. IBD: inflammatory bowel disease; RA: rheumatoid arthritis; T1DM: type 1 diabetes mellitus.

Autoimmune diseases are a series of related diseases characterized by dysregulation of the immune system, in which immune cells cannot distinguish self-antigens from non–self-antigens and give rise to activation of immune cells to attack autoantigens, leading to inflammation and multi-tissue damage [28]. T1DM, RA, and IBD are autoimmune diseases [29]. Moreover, some of the autoimmune diseases are organ-specific, such as primary biliary cirrhosis, and some reflect a variety of immunological dysfunctions involving multiple organ immune dysfunction such as systemic lupus erythematosus [30]. T1DM is dramatically increasing in many parts of the world [31], they affect millions of people and are prone to serious complications such as heart and kidney disease, stroke, and blindness [32] (Figure 2). RA is an autoimmune disease that damages joints and other tissues and organs such as the heart, kidneys, lungs, digestive system, eyes, skin, and nervous system [33]. IBD is a chronic, progressive immune-mediated inflammatory condition of the gastrointestinal tract [34]. The incidence of IBD has rapidly increased worldwide. Patients with IBD may suffer from malnutrition, characterized by an energy or nutrient imbalance, leading to sarcopenia, micronutrient deficiencies, overweight, obesity, and sarcopenic obesity [35]. Sarcopenia is a common complication of autoimmune diseases, and the primary cause is the loss and damage of skeletal muscle induced by immune dysfunction [36-38].

## Type 1 Diabetes Mellitus and Sarcopenia

T1DM results from the autoimmune dysfunction and death of β-cells, requiring lifelong insulin therapy [39]. This condition arises from a complex interaction between invading or resident macrophages and T cells. These immune cells release various substances, such as chemokines and cytokines, in the islet microenvironment that promote the apoptosis of beta cells. Simultaneously, β-cells can attract and activate immune cells to the islet area through their signaling, especially in response to stress, damage, or cell death [40]. The primary consequence of T1DM is hyperglycemia. Research indicates that the insulin-like growth factor (IGF) and IGF-binding protein (IGFBP) axis are crucial in maintaining normal glucose levels. Patients with T1DM showed a significant reduction in serum IGF-I levels. Additionally, mitophagy plays a crucial role in regulating the autoimmune response during the development of T1DM by preventing the accumulation of defective or dysfunctional mitochondria in pancreatic cells [41]. Muscle fibers can be damaged and undergo adverse remodeling due to the hyperglycemic and inflammatory microenvironment caused by T1DM, ultimately leading to sarcopenia [42]. A cross-sectional study showed that the prevalence of sarcopenia in participants aged 65 years or older with T1DM is 42.9%, which is significantly higher than that in people of the same age without sarcopenia [43]. Moreover, musculoskeletal system health would be affected if T1DM was poorly controlled in childhood and adolescence, and then sarcopenia would become the late complication of T1DM in adults [42].

Skeletal muscle is a primary driver organ that maintains whole-body glycemic balance [44]. Poor blood glucose control is the main risk factor for T1DM, which is complicated by sarcopenia [45]. IGFs, especially IGF1 and IGF2, can promote glucose metabolism, with their availability regulated by IGFBPs. In sum, IGFs are dysregulated both before and after the clinical diagnosis of T1DM and may serve as novel biomarkers to improve disease prediction [46]. Subsequently, Hata et al [47] discovered that a low serum IGF-1 level is associated with sarcopenia and low skeletal muscle mass in subjects with T1DM. Moreover, growth hormone could regulate cell survival and the musculoskeletal system function through growth hormone/IGF1 signaling pathway [48]. Other researchers found that IGF1 activates the AKT/PKB pathway and protein synthesis pathway by binding to the IGF1 receptor, thereby increasing muscle synthesis [49]. In addition, previous reports have pointed out that mitochondrial dysfunction is the common cause of regulating muscle degeneration induced by aging and T1DM [50]. However, increased oxidative stress, decreased respiration and oxidative capacity, and increased permeability pore opening in mitochondria could promote the process of apoptosis.

## Rheumatoid Arthritis and Sarcopenia

RA is an autoimmune disease characterized by the presence of symmetrical polyarthritis, which tends to lead to severe cartilage and bone destruction, thereby resulting in joint pain and arthritis [51]. Persistent joint inflammation can lead to cartilage and bone damage, joint damage, and even disability if the patients receive treatment not in time [52]. The pathogenesis of RA is complex and has not been fully elucidated [53]. Genetic susceptibility contributes to the increased prevalence of RA pathogenesis [54]. Epigenetic factors, such as DNA methylation and histone acetylation play a key role in RA [55]. The production of autoantibodies and the presence of autoreactive T cells in blood and synovial structures are distinguishing features of RA [56].

Studies have shown that RA is a potentially pathogenic effect of low appendicular lean mass and low grip strength [49]. The injury of RA to skeletal muscle can accelerate the occurrence of sarcopenia and seriously affect the life quality of patients [57,58]. Li et al [59] pointed out that the prevalence of sarcopenia in patients with RA was 31%, which is much higher than that in people without RA. Another cross-sectional study of 388 patients with RA showed that 37.1% of the participants had sarcopenia (14.7% severe sarcopenia, 22.4% sarcopenia) and 49.0% had low muscle mass [60]. Even more importantly, the incidence of sarcopenia increases with the lastingness of RA progression [59,61]. It is worth noting that RA patients with a higher percentage of body fat are more likely to develop sarcopenia [62].

Chronic inflammation is closely related to muscle damage in RA [63]. RA primarily affects the joints, but the risk and susceptibility factors can also cause inflammation in mucosal sites such as the mouth, lungs, and intestines. These sites can be recognized by the adaptive immune system, which triggers an immune response with the help of antigen-presenting cells. This response occurs in secondary lymphoid tissues, resulting in the production of autoantibodies. Subsequently, activated stromal cells like fibroblast-like synovial cells, antigen-presenting cells, and macrophages (MΦ) produce various inflammatory cytokines, including interleukin (IL)-1, IL-2, IL-6, IL-8, IL-17, tumor necrosis factor-α (TNF-α), IFNγ (interferon gamma), and others, which can cause local inflammation and damage [64]. Researchers indicated that skeletal muscle metabolic disorders might be the leading cause of RA combined with sarcopenia [10]. However, skeletal muscle metabolism disorders can lead to loss of skeletal muscle due to impaired muscle anabolism, excessive muscle catabolism, or a combination of both [65]. The ubiquitin-proteasome pathway is a key pathway to increase muscle proteolysis [62,63]. Excessive production of proinflammatory factors makes a significant contribution to the breakdown of muscle protein. Higher levels of TNF-α and IL-1β induced by RA condition could elevate the expression level of IGFBP-5, IGFBP-3, scroggin-1, and muscle ring finger protein-1 (MuRF-1) through regulating the ubiquitin-proteasome system, thereby leading to increased hydrolysis of myofibrillar and soluble proteins [65].

Additionally, it was found that the amount of body fat and the density of muscle were closely linked to disability and physical performance in patients with RA. Specifically, individuals with a higher percentage of body fat showed a stronger correlation with the development of sarcopenia in RA. Therefore, it is recommended that the focus be on reducing fat and improving muscle quality as effective approaches to alleviating the disability of patients with impaired physical functioning [62]. Exercise could increase muscle mass in patients with RA effectively, so physical inactivity might be another risk factor for the high prevalence of RA combined with sarcopenia [66].

## Inflammatory Bowel Disease and Sarcopenia

IBD, which includes ulcerative colitis and Crohn disease, is a progressive immune-mediated bowel disease for which there is no effective therapy treatment currently [57,67]. The intestinal microecology of patients with IBD is disturbed, which triggers changes in host immune response and metabolism that subsequently promote inflammation. The inflammatory response to IBD is characterized by a sustained increase in the production of inflammatory cytokines by T cells (TNF-α, IL-6, IL-1b, etc) [68]. Increased IL-12 ⁄ 23 and IFN-c levels, mediated by T helper cell 1, are supposed to be major factors in the maintenance of inflammation in patients with Crohn disease [69]. But T helper cell 2-mediated IL-5 and IL-13 levels elevation is an important factor for inflammation in ulcerative colitis patients [70].

Chronic inflammation and malnutrition resulting from IBD contribute to the development of sarcopenia [71,72]. Sarcopenia is a common condition in IBD patients and it increases fatigue and decreased quality of life. According to a recent study, sarcopenia occurs in 52% of people with Crohn disease and 37% of people with ulcerative colitis [71]. Although the underlying mechanism of sarcopenia in IBD patients remains unclear, studies indicated that the gut-skeletal muscle axis sheds light on the pathophysiology of IBD-associated sarcopenia. This axis comprises specific mediators from the gut that activate signaling pathways for amyotrophic and antimuscular atrophy in skeletal muscle cells [73]. The up-regulation of TNF-α, NF-κB (nuclear factor kappa-B), and JAK/STAT signaling pathways is an important factor in the pathogenesis of sarcopenia [74]. Studies have shown increased levels of proinflammatory factors such as TNF-α, IL-6, circulating lipopolysaccharide, antimyogenic mediators, and promyogenic mediators (myostatin and insulin-like growth factor-1) in IBD patients [75,76]. Both TNF-α and IL-6 can activate NF-κB, which induces transcription of components of the ubiquitin-proteasome proteolytic pathway, such as atrophic protein-1 and MuRF-1, triggering protein degradation [77]. In addition, NF-κB further enhances IL-6 expression, creating a vicious cycle of inflammation and muscle protein degradation [78]. Inhibiting myostatin and muscular dystrophy factors such as atrophin-1 and MuRF-1 and enhancing myogenic factors including MyoG (‌myogenin) and MyoD (myoblast determination) can improve muscle atrophy [79]. Notably, the JAK/STAT signaling pathway is highly involved in the pathogenesis of IBD [80], mediating the function of several inflammatory cytokines involved in intestinal inflammation, such as IL-2, IL-4, IL-6, IL-7, IL-9, IL-12, IL-15, IL-21, IL-23, and IFN-γ [81].

Malnutrition often occurs in patients with intestinal insufficiency and intestinal failure. Malabsorption of micronutrients and macronutrients resulting from malnutrition can cause changes in body composition and increase the risk of sarcopenia [82]. In general, dietary patterns would have a great influence on the gut microbiota structure and function of patients with inflammatory diseases. For instance, the Mediterranean diet is beneficial for IBD patients with reduced inflammation and improved immune function, but the Western diet has the opposite effect [83]. In addition, vitamin D is involved in regulating muscle cell proliferation, differentiation, and regeneration. Consistent with this, research has shown that vitamin D supplementation is expected to reduce the occurrence of sarcopenia in IBD patients [84]. The inability to maintain protein balance after meals can lead to a decrease in muscle mass in patients with Crohn disease, exacerbating the occurrence of sarcopenia [85].

## Ethical Considerations

This study used exclusively publicly accessible deidentified datasets, ensuring the absence of personal identifiers or sensitive information. Therefore, no ethical approval or declaration was required per the journal’s policy and institutional guidelines. Given the absence of secondary analysis involving pre-existing nonidentifiable data, the research maintained full alignment with the Declaration of Helsinki principles. Methodological rigor was further reinforced through strict adherence to ethical protocols outlined in the JMIR Editorial Policies for data governance, as well as compliance with internationally validated open-science frameworks for responsible data use.

## Discussion

**Figure 3.**
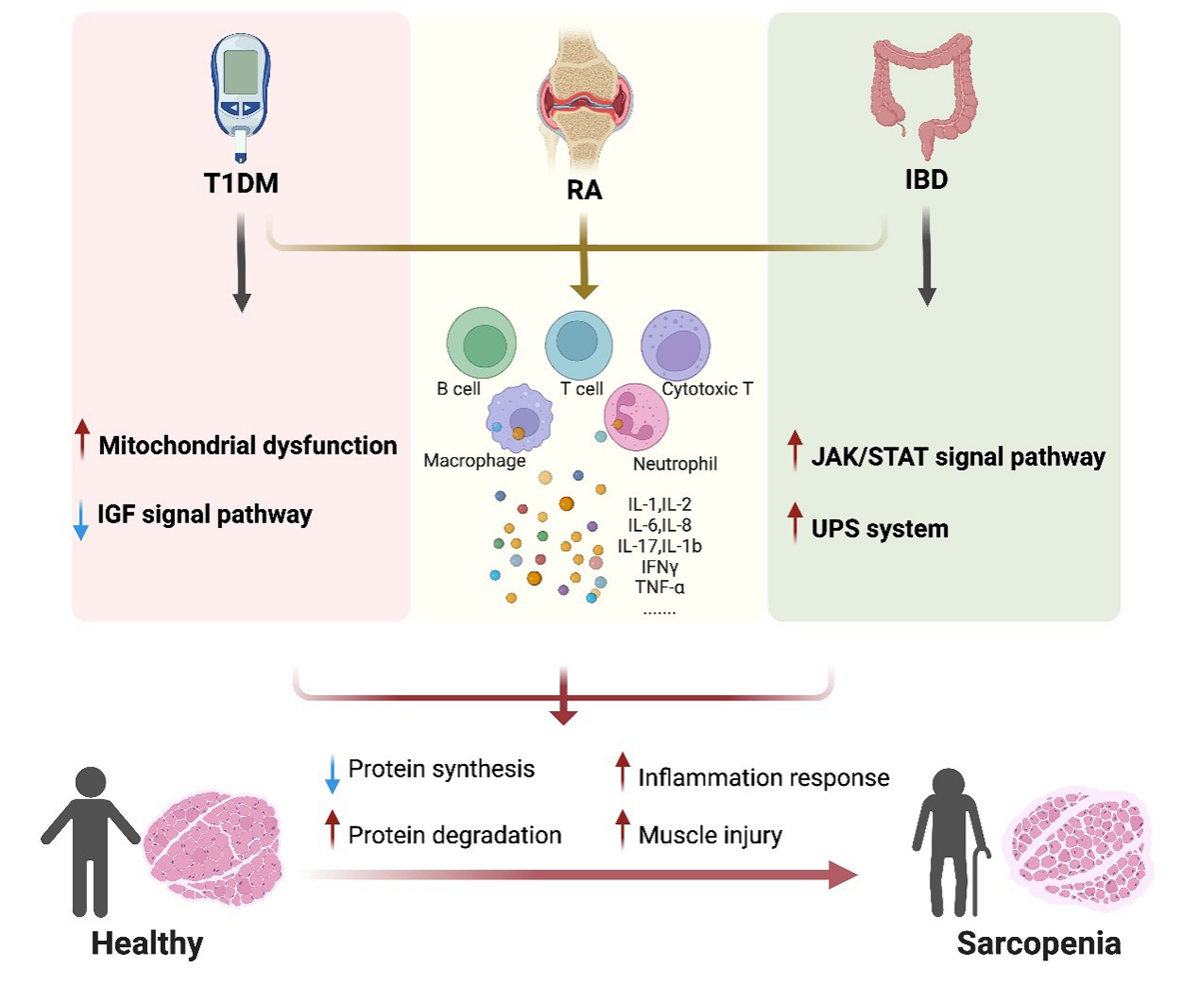
The potential mechanism of autoimmune diseases combined with sarcopenia. IBD: inflammatory bowel disease; IFN: interferon; IGF: insulin-like growth factor; IL: interleukin; JAK/STAT: Janus kinase/signal transducer and activator of transcription; RA: rheumatoid arthritis; T1DM: type 1 diabetes mellitus; TNF: tumor necrosis factor; UPS: ubiquitin proteasome system.

Sarcopenia is common in the 3 autoimmune diseases commented on in this manuscript, T1DM, RA, and IBD. The combination of autoimmune diseases and sarcopenia can result in a diminished life quality, long-term disease prognosis management, and impose a substantial financial burden. Thus, it is important to investigate the relationship between sarcopenia and autoimmune diseases. Essentially, perturbations within the immune microenvironment of patients with autoimmune disorders elicit fluctuations in the populations of immune cells and alterations in the expression patterns of relevant factors. These modifications subsequently disrupt the functional coherence of muscle regeneration signaling cascades, ultimately underlying the pathogenesis of sarcopenia (Figure 3).

Autoimmune diseases are closely associated with alterations in the immune microenvironment. Pathological interactions between T-cells and B-cells serve as the foundation for numerous autoimmune diseases [86]. For T1DM [87] and RA [88], disruption of T-cell and B-cell tolerance, along with the production of autoantibodies, typically precede the diagnosis of autoimmune diseases. T1DM is characterized as the pancreatic beta cells destroyed by the immune system, resulting in life-long use of exogenous insulin, which places a great burden on patients and medical resources [89]. Pancreatic β cell injury is primarily attributed to T cells while antigen-presenting B cells exacerbate this damage by presenting antigens to T cells [90]. Multiple pieces of evidence indicate that T cells play a pivotal role in driving autoimmune responses in RA, with an abundance of these cells observed within inflamed synovial membranes [91]. The intestinal environment is highly complex, and the immune system maintains the entire intestine’s health [92]. Among various factors contributing to the balance of intestinal microbiota, B cells undergo activation and maturation to generate the largest population of plasma cells secreting immunoglobulin A within the human body, thereby promoting immune system homeostasis [93]. Additionally, Follicular helper T cells are enriched in several inflammatory conditions, particularly within the intestine where they exhibit higher levels compared with other tissues [94].

Muscle atrophy represents a significant contributor to sarcopenia development wherein immune cells play a vital role in muscle regeneration. Immune cells release key inflammatory mediators that regulate the muscle fiber microenvironment [95]. Early recruitment of neutrophils leads to robust nonspecific responses that further exacerbate muscle fiber and extracellular matrix injuries [96-98]. Neutrophils exacerbate cellular injury by releasing reactive oxygen species, which induce muscle fiber damage and increase extravasated vascular permeability [99]. Furthermore, neutrophils secrete proinflammatory cytokines IL-6, IL-1β, and TNF-α, promoting satellite cell proliferation while inhibiting differentiation [100]. Both IL-6 and reactive oxygen species have been demonstrated to cause mitochondrial dysfunction/damage and alterations in protein stability within muscle fibers [99,100].

Cytotoxic T lymphocytes exhibit similar functions to neutrophils during the early stages of injury response [101]. Additionally, neutrophils and cytotoxic T lymphocytes recruit inflammatory macrophages to aid in the phagocytosis of damaged tissue for clearance while releasing proinflammatory mediators that stimulate satellite cell and fibroblast proliferation and enhance vascular permeability [102]. However, the investigation of their roles in patients with autoimmune diseases complicated by sarcopenia remains limited. It can be speculated that these disruptions within the skeletal muscle microenvironment may lead to increased loss of muscle mass and fibrosis, resulting in reduced muscle plasticity, weakness, and fatigue.

## Conclusions

Autoimmune diseases are closely associated with alterations in the immune microenvironment which plays a crucial role in skeletal muscle differentiation and remodeling [103]. The pathogenesis of autoimmune diseases combined with sarcopenia can be attributed to an imbalance of hormones, growth factors, and proinflammatory factors in the immune microenvironment. This leads to muscle metabolism disorders resulting in a decline in muscle mass due to blocked muscle synthesis and excessive muscle catabolism. To mitigate muscle loss, it is recommended to improve diet quality by consuming nutrients like vitamin D [104] and high-quality protein supplements [105]. Additionally, engaging in physical exercise may be an effective therapy to prevent autoimmune diseases combined with sarcopenia. However, there is still a lack of effective clinical treatment for these diseases, and the pathogenesis of autoimmune diseases combined with sarcopenia needs to be further elucidated [66,106,107]. Future research should focus on understanding the molecular mechanisms of various autoimmune diseases combined with sarcopenia and developing targeted therapies [108].

## Abbreviations

BIA: bioelectrical impedance analysis
DXA: dual-energy x-ray absorptiometry
IBD: inflammatory bowel disease
IFNγ: interferon gamma
IGF: insulin-like growth factor
IGFBP: insulin-like growth factor–binding protein
IL: interleukin
JAK/STAT: Janus kinase/signal transducers and activators of transcription
MuRF-1: muscle ring finger protein-1
MyoD: myoblast determination
MyoG: myogenin
NF-κB: nuclear factor kappa-B
RA: rheumatoid arthritis
T1DM: type 1 diabetes mellitus
TNF-α: tumor necrosis factor-α
